# The emerging importance of lymphatics in health and disease: an NIH workshop report

**DOI:** 10.1172/JCI171582

**Published:** 2023-09-01

**Authors:** Babak J. Mehrara, Andrea J. Radtke, Gwendalyn J. Randolph, Brianna T. Wachter, Patricia Greenwel, Ilsa I. Rovira, Zorina S. Galis, Selen C. Muratoglu

**Affiliations:** 1 Department of Plastic and Reconstructive Surgery, Memorial Sloan Kettering Cancer Center, New York, New York, USA.; 2 Lymphocyte Biology Section and Center for Advanced Tissue Imaging, Laboratory of Immune System Biology, National Institute of Allergy and Infectious Diseases, NIH, Bethesda, Maryland, USA.; 3 Department of Pathology and Immunology, Washington University School of Medicine, St. Louis, Missouri, USA.; 4 Laboratory of Clinical Immunology and Microbiology, National Institute of Allergy and Infectious Diseases, NIH, Bethesda, Maryland, USA.; 5 Division of Digestive Diseases & Nutrition, National Institute of Diabetes and Digestive and Kidney Diseases, and; 6 Division of Cardiovascular Sciences, National Heart, Lung, and Blood Institute, NIH, Bethesda, Maryland, USA.

## Abstract

The lymphatic system (LS) is composed of lymphoid organs and a network of vessels that transport interstitial fluid, antigens, lipids, cholesterol, immune cells, and other materials in the body. Abnormal development or malfunction of the LS has been shown to play a key role in the pathophysiology of many disease states. Thus, improved understanding of the anatomical and molecular characteristics of the LS may provide approaches for disease prevention or treatment. Recent advances harnessing single-cell technologies, clinical imaging, discovery of biomarkers, and computational tools have led to the development of strategies to study the LS. This Review summarizes the outcomes of the NIH workshop entitled “Yet to be Charted: Lymphatic System in Health and Disease,” held in September 2022, with emphasis on major areas for advancement. International experts showcased the current state of knowledge regarding the LS and highlighted remaining challenges and opportunities to advance the field.

## Introduction

In September 2022, the NIH convened the “Yet to be Charted: Lymphatic System in Health and Disease” workshop (1, 2) to highlight the role of the lymphatic system (LS) in health and disease and identify gaps and opportunities in lymphatic research. Here, we provide a high-level overview of some discussions of interest, including a summary of lymphatic development and function, with examples of exciting areas of study to encourage new investigators to enter the field.

## History of lymphatic research

**Figure 1:**
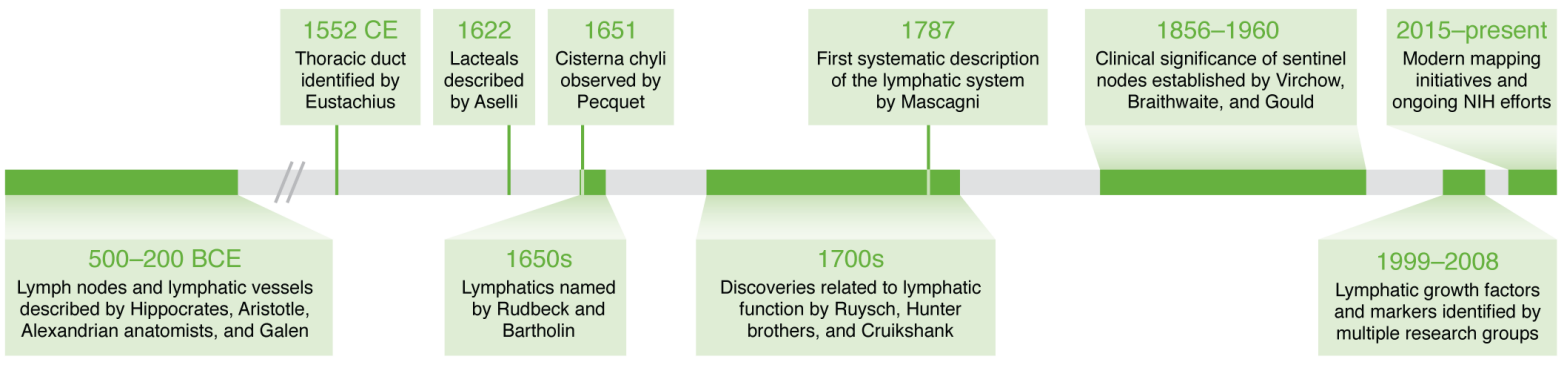
Key milestones punctuate the centuries-long pursuit to map the lymphatic system. Ten important events in the history of lymphatic research that occurred between 500 BCE and the present day. See Supplemental Table 1 for a comprehensive timeline of lymphatic discoveries and NIH-led activities and funding opportunities.

The first description of “lymphatic glands swelling” is attributed to an Egyptian hieroglyph circa 1600 BCE (3–5). Since then, the long arc of lymphatic discovery has been punctuated by substantial contributions from the ancient Greeks, famed physician-artists of the Renaissance, anatomists of the Enlightenment, and present-day scientists (Figure 1 and Supplemental Table 1; supplemental material available online with this article; https://doi.org/10.1172/JCI171582DS1). A survey of the literature (4, 6–8) reveals several distinguishing themes that continue to unite researchers across the epochs. First and foremost, past and present investigators possess a relentless drive to visualize the LS, describe its function, and define morphological and molecular features of the system and its parts. Another hallmark is the ambitious quest to create a comprehensive lymphatic atlas, from the solo pursuit of Paolo Mascagni in 1784, who is credited with mapping 50% of all lymphatic vessels of the human body (7), to international efforts exploring the human body at single-cell resolution (9, 10). As with all scientific progress, LS knowledge has advanced at the pace of technical innovation, as demonstrated first by the application of microscopy to medicine by Malpighi in the 1600s (11) and more recently by use of dynamic contrast–enhanced magnetic resonance lymphangiography (DCMRL) to understand central lymphatic anatomy.

**Figure 2:**
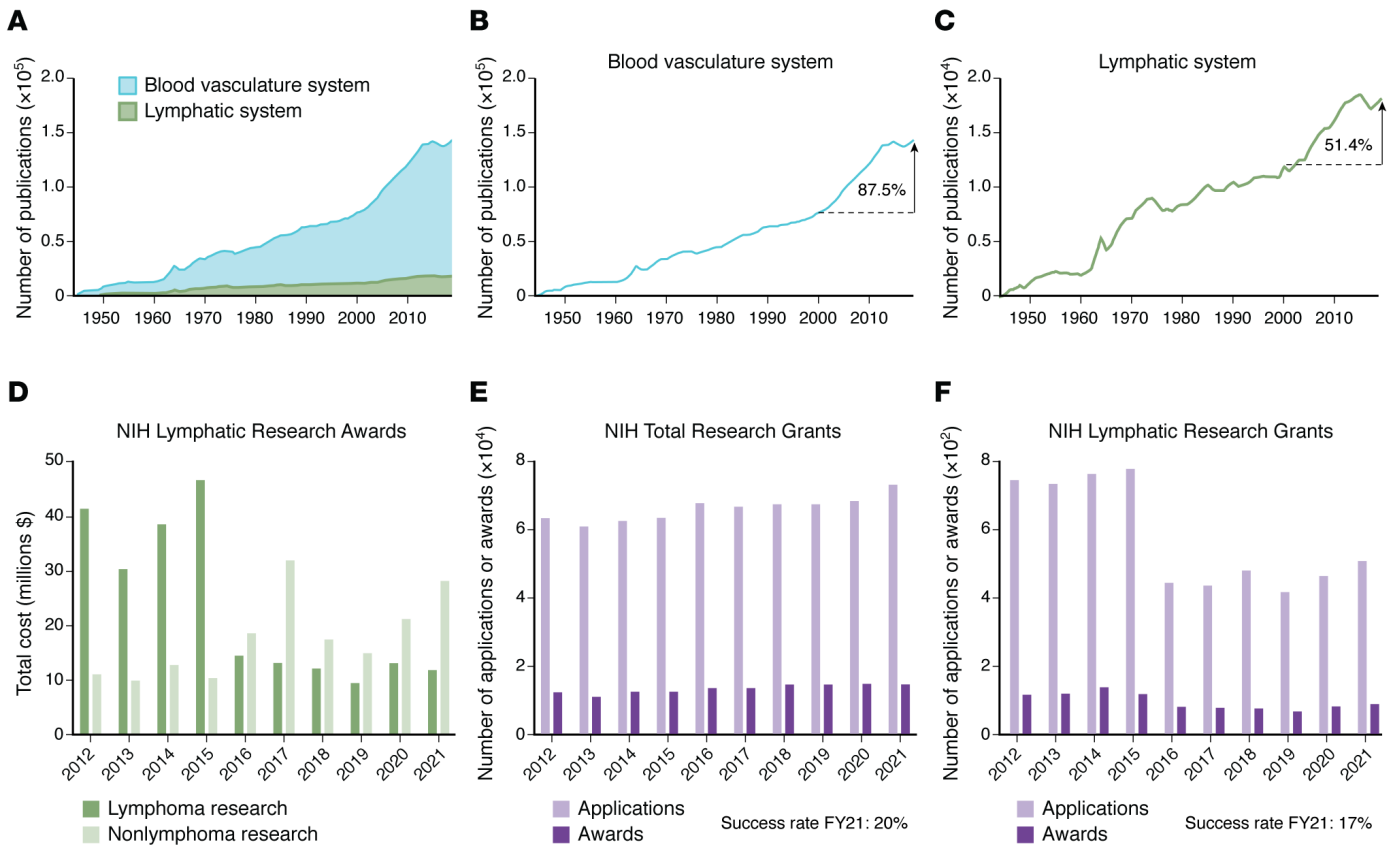
Publications and NIH funding data summarize trends for lymphatic research. (**A**–**C**) Bibliometric analysis from 1944 to 2019 used the PubMed Advanced Search Builder and Medical Subject Headings (MeSH) terms, accessed on March 23, 2023. Blood vasculature search queries included ((“Cardiovascular Diseases”[MeSH Terms] OR (“Cardiovascular System”[MeSH Terms] AND (“Mice”[MeSH Terms] OR “Humans”[MeSH Terms])) OR “Blood Vessels”[MeSH Terms] OR “Blood”[MeSH Terms]) OR “Heart” [MeSH Terms]). Lymphatic system queries included lymphatic anomalies: ((“Lymphatic Diseases”[MeSH Terms] OR (“Lymphatic System”[MeSH Terms] AND (“Mice”[MeSH Terms] OR “Humans”[MeSH Terms])) OR “Lipedema”[MeSH Terms] OR “Lymphedema”[MeSH Terms] OR “Lymphatic Vessels”[MeSH Terms] OR “Lymphoid Tissue”[MeSH Terms])). (**B** and **C**) The percentage increase was calculated using the following formula: [(no. publications 2019 – no. publications 2000)/no. publications 2000] × 100. (**A**) Area graphs showing blood vasculature and lymphatic system from 1944 to 2019. (**B**) Line graphs correspond to the number of blood vasculature system publications and show the growth across the last 75 years. The number of publications increased by 87.5% in the last 20 years. (**C**) Comparatively, the number of lymphatic system publications has a growth rate of 51.4% for the last 20 years. (**D**–**F**) Portfolio analysis of NIH lymphatic research grant applications and awards from 2012 to 2021 using the RCDC categories “lymphatic research,” “lymphoma,” and “lymphedema” indicates total NIH funding amounts. The analysis was performed by the National Heart, Lung, and Blood Institute’s Office of Planning, Analytics, and Evaluation on August 23, 2022. (**D**) Total cost of NIH-funded lymphatic research awards; lymphoma (dark green bars) and nonlymphoma (light green bars) lymphatic research awards. (**E**) Number of all NIH research grant applications (light purple bars) and awards (dark purple bars), including competing applications and awards. The success rate for fiscal year (FY) 2021 of 20%. (**F**) Number of NIH lymphatic research grant applications (light purple bars) and awards (dark purple bars), including first-time and competing applications. The success rate for awards in FY2021 was 17%.

The body-wide reach of the LS has long been appreciated, with groundbreaking discoveries related to organ-specific lymphatic networks appearing in the 18th and 19th centuries (Supplemental Table 1). Despite these impressive achievements, the LS remains one of the last frontiers of anatomical medicine, exemplified by the recent discovery of the glymphatic system and meningeal lymphatic network in the central nervous system (CNS) (12–15). Although the lymphatic vasculature is outpaced by the blood vasculature as a subject of publications (Figure 2, A–C), the last two decades demonstrate a growing field (Supplemental Table 1 and Figure 2, A–C), a renewed commitment to education and outreach through international workshops (Supplemental Table 1), and a substantial investment in lymphatic research by the NIH (Figure 2, D–F).

## Lymphatic anatomy and function: a brief overview

The LS is composed of lymphoid organs distributed throughout the body and lymphatic vessels that transport interstitial fluid (ISF), antigens, lipids, cholesterol, immune cells, and other materials. Beyond recognition as critical regulators of fluid homeostasis, a growing literature now establishes lymphatic vessels as mediators of immune cell trafficking and immune interactions, absorption of nutrients, and clearance of inflammation. Lymphatic vessels usually run alongside major blood vessels and are present in most organs. Initial lymphatic vessels, or lymphatic capillaries, are single-cell-layered, blind-ended vessels with lymphatic endothelial cells (LECs) arrayed in an overlapping manner. These vessels possess loose intracellular junctions that allow influx of cargo. This arrangement is termed a “button” junction and refers to open areas between points of adhesion between LECs (16). LECs are also attached to the extracellular matrix by anchoring filaments that stabilize the vessels for sustained openings under tension, thus facilitating influx of cargo and responsiveness to tissue dynamics. LECs can also actively transcytose large molecules into the capillary lymphatic lumen (17, 18). Transcytosis may play a key role in regulating immune responses by sequestering or archiving antigens (19, 20).

Capillary lymphatics drain into progressively larger vessels called collecting lymphatics that have a smooth muscle cell coverage and are made up of individual units termed “lymphangions” bound by a proximal and a distal valve. Lymphatic muscle cell (LMC) contraction is regulated by ion channels, can be modulated by a variety of stimuli, and occurs intrinsically and spontaneously (21, 22). Valves are arranged anatomically to regulate lymphatic flow toward the heart. Coordinated contraction and relaxation of LMCs allow each lymphangion to fill and empty, promoting lymph flow against a progressively higher downstream pressure in the blood vessels. Skeletal muscle contraction and arterial pulsation contribute to lymphatic flow as discussed below.

Afferent collecting lymphatics drain into lymph nodes where antigens and immune cells are filtered and sampled by lymph node–resident cells (23–25). Fluid from the lymph nodes is drained by efferent lymphatics that drain into successively larger collecting lymphatic channels. ISF from the lower extremities, trunk, left chest, and left upper extremity drains into the thoracic duct, which empties back into the circulatory system via the left subclavian vein. Lymph drainage from the head, right arm, and right chest drains into the right lymphatic duct, which drains into the right subclavian vein. There is marked variability in the anatomy of the thoracic duct in humans (26, 27). In humans, it is estimated that 8–12 liters of ISF is produced per day and returned to the blood vasculature via the LS and absorption in lymph nodes. After nodal reabsorption on average, 4 liters of lymph fluid is drained by the efferent LS back into the systemic circulation every 24 hours (28). A revised model of Starling forces related to cardiac lymphatic function underscores the contribution of lymphatic vessels to fluid balance (29).

## Lymphatic development

Lymphatic vessels develop from embryonic venous blood endothelial cells (BECs) that line the anterior surface of the cardinal vein (30–33). This process is initiated by induction of the transcription factor prospero homeobox 1 (PROX1) in venous BECs by sex-determining region Y box 18 (SOX18) and chicken ovalbumin upstream promoter transcription factors (COUP-TFII). *Prox1* specifies lymphatic endothelial fate, and *Prox1*-knockout mice completely lack lymphatics and fail to survive (30, 34). LECs that sprout from the cardinal vein express α_9_ integrin and VEGFR3 and migrate toward mesenchymal cells via VEGFC gradients (35). VEGFC is required for the emergence and migration of LECs, and its activity is modulated by proteolytic cleavage by the matrix protein collagen and calcium binding EGF domains 1 (CCBE1) and the metalloproteinase, a disintegrin and metalloproteinase with thrombospondin motifs 3 (ADAMTS3).

The developmental origins of the BECs in the cardinal vein that differentiate into LECs have been a subject of intense study. The discovery that lymphatic tissue could have nonvenous origins shifted the paradigm for the field and was first appreciated with regard to dermal and cardiac lymphatics (36–38). Recent studies in mice using lineage tracing suggest that cells from the paraxial mesoderm preferentially differentiate into the BECs on the dorsal aspect of the cardinal vein and indicate that this process precedes expression of lymphatic markers and formation of the lymphatic network in the heart, lung, skin, and meninges (39). Conditional *Prox1* deletion in cells of the paraxial mesoderm results in an absence of dermal lymphatics in the lumbar region of embryonic day 15.5 embryos with subcutaneous edema and blood-filled lymphatic vessels in the cervical and thoracic skin. Recent experiments using single-cell RNA analysis and lineage tracing suggest that LECs may arise directly from paraxial mesoderm–derived lymphangioblasts (40). Whether paraxial mesoderm cells first differentiate into BECs in the anterior cardinal vein and then develop into LECs, and whether paraxial mesoderm–derived cells differentiate into specialized angioblasts and then into LECs, remain debated topics that require further study.

Lymphatic valves develop from lymphatic valve progenitor cells that grow into the vessel lumen and encapsulate an extracellular matrix core in a complex developmental process. This process is regulated by the transcription factors PROX1, GATA2, FOXC2, and β-catenin (41–45). Oscillatory shear stress upregulates these transcription factors, as well as the shear-responsive transcription factors KLF2 and KLF4 (46). VE-cadherin is required for mechanotransduction signaling that upregulates these transcription factors. β-Catenin is a VE-cadherin binding partner, and β-catenin signaling was shown to be required for the specification of valve territories during embryonic valve development (47). Recently, FOXO1 was discovered as the first repressor of the lymphatic valve gene program (48). Thus, FOXO1 deficiency induced the upregulation of many valve genes, including *Foxc2*, *Gata2*, *Klf4*, and *Klf2*. FOXC2 is essential for lymphatic valve development and maintenance, and heterozygosity for *Foxc2* causes lymphedema distichiasis (49, 50). Interestingly, deletion of the *Foxo1* gene was able to fully rescue the loss of valves in *Foxc2*-heterozygous mice, suggesting translational potential for this protein.

How LMCs develop and coordinate pumping of collecting lymphatic vessels in normal and diseased states is an important frontier for lymphatic research. Recruitment of muscle cells is regulated by PDGFB and angiopoietin-2, and by semaphorin/neuropilin signaling via semaphorin 3A (SEMA3A) and neuropilin 1 (NRP1) (51, 52). Emerging methods for selective deletion of LMC target genes will facilitate future research endeavors (53).

## Lymphatic dysfunction

**Figure 3:**
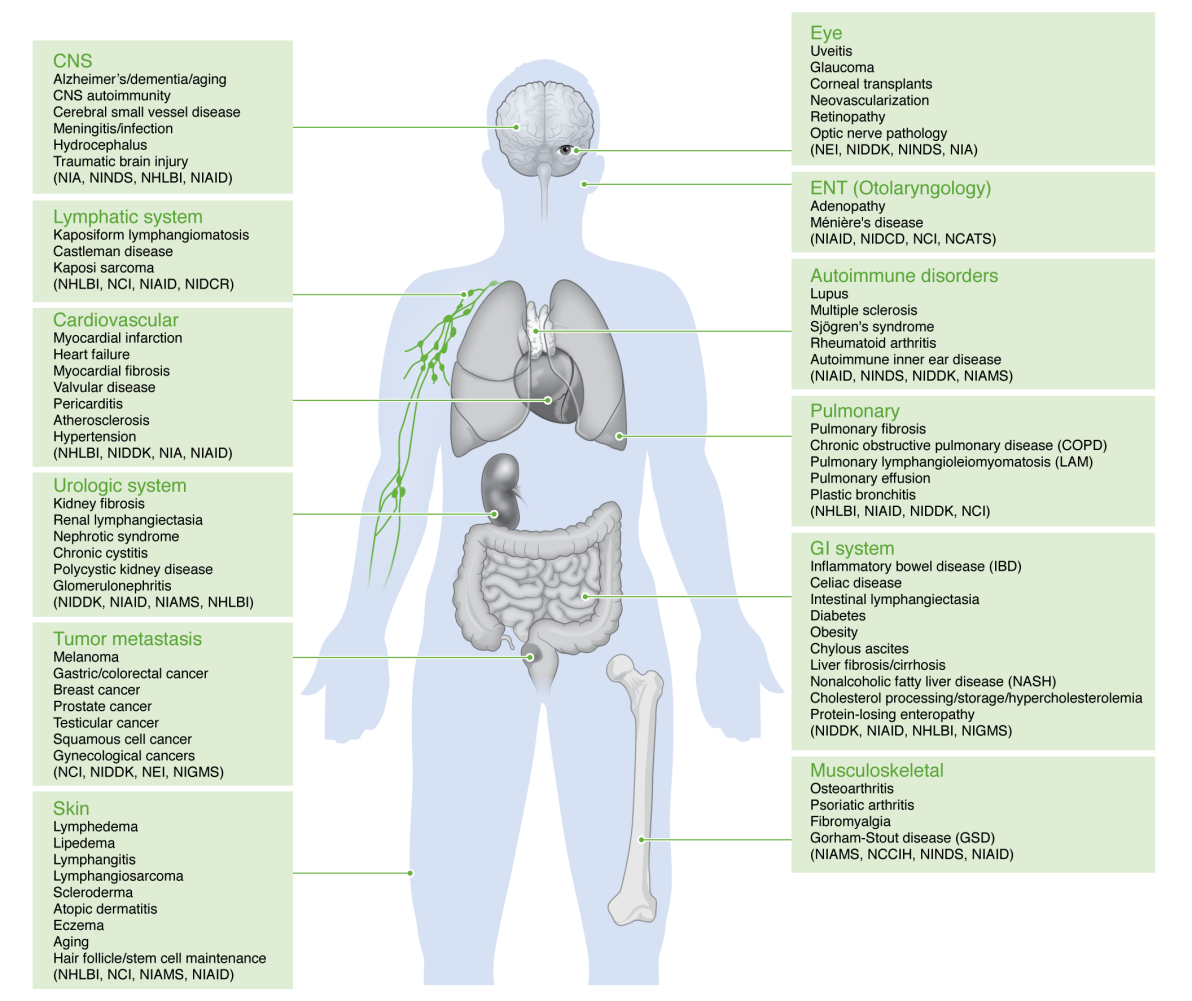
Lymphatic diseases manifest across the human body. Changes in normal lymphatic function can alter multiple systems in the body and manifest as different and varied pathologies. There are many conditions related to lymphatic dysfunction within all body systems. Hematological malignancies were not included because the focus of the workshop was on understudied diseases of the lymphatic system. Various NIH Institutes or Centers that currently fund lymphatic research are indicated: NCATS, National Center for Advancing Translational Sciences; NCCIH, National Center for Complementary and Integrative Health; NCI, National Cancer Institute; NEI, National Eye Institute; NHLBI, National Heart, Lung, and Blood Institute; NIA, National Institute on Aging; NIAID, National Institute of Allergy and Infectious Diseases; NIAMS, National Institute of Arthritis and Musculoskeletal and Skin Diseases; NIDCD, National Institute on Deafness and Other Communication Disorders; NIDCR, National Institute of Dental and Craniofacial Research; NIDDK, National Institute of Diabetes and Digestive and Kidney Diseases; NIGMS, National Institute of General Medical Sciences; NINDS, National Institute of Neurological Disorders and Stroke.

Lymphatic abnormalities play a key role in many pathological processes (Figure 3). Here, we highlight a few disease conditions but exclude hematological malignancies, reflecting the focus and aims of the workshop. Comprehensive reviews on organ-specific LS and its contributions to health and disease have been published (29, 54, 55).

### CNS lymphatic dysfunction.

The mechanisms by which waste and metabolites are cleared in the CNS is a topic of intense study. Indeed, the existence of functional lymphatic vessels in the meninges was only recently described (56). A recently named “glymphatic” pathway may mediate waste removal from the brain. The system includes the perivascular space and glial cells (astrocytes), but not formal lymphatic vessels. Cerebrospinal fluid (CSF) is produced by epithelial cells in the choroid plexus, surrounds the brain, and circulates within the subarachnoid space. The glymphatic system removes intercellular metabolites, waste products, proteins, and antigens from the brain through CSF-ISF exchange along channels formed between the vein walls and the astrocytic end feet (12, 57). This process is regulated by pulsation of brain arteries that drive CSF through astrocyte end feet across the brain parenchyma (57, 58). Lymphatic vessels in the dura mater surrounding the brain transport CSF waste products and inflammatory cells into the deep cervical lymph nodes (56, 59).

The glymphatic system is influenced by aging, sleep cycles, and genetic factors (58, 60–62). Preclinical studies indicate that dysfunction of the glymphatic system, from aging or injury, may contribute to the development of neurological disorders like Alzheimer’s disease, Parkinson’s disease, small-vessel cerebrovascular disease, and traumatic brain injury (63–66). Compared with those in young, healthy individuals, aging meningeal lymphatic vessels have decreased diameter and specific branching patterns and drain to cervical lymph nodes, potentially imperiling upstream glymphatic function. In Alzheimer’s disease, impaired function of the glymphatic system may diminish waste clearance and thereby allow accumulation of toxic protein aggregates. Strategies to improve meningeal lymphatic function, e.g., treatment with VEGFC, improve learning and memory tests in older mice, likely via enhanced lymph drainage (67). Notably, ligation of meningeal lymphatics eliminated these benefits (67).

In other CNS-related diseases, the transport of self-antigens via the CNS LS is thought to initiate autoimmune responses in draining lymph nodes. Similarly, antitumor immune responses were inhibited by deep cervical lymph node resection or enhanced by increasing lymphatic function using VEGFC injections (68). Thus, modulators of lymphatic function may serve as therapies or may increase the efficacy of existing treatments for CNS disorders and aggressive brain tumors.

### Cardiac lymphatic system dysfunction.

Given the indispensable role of lymphatics in maintaining tissue fluid homeostasis, immune cell trafficking, reverse cholesterol transport, and nutritional lipid uptake, the LS function has been implicated in several cardiovascular pathologies, e.g., atherosclerosis, myocardial infarction, and heart failure (69).

Unlike several other tissues, cardiac lymphatics depend on myocardial contractions to propel lymphatic flow. Additionally, the rate and force of cardiac contractions substantially affect lymph flow (70). Insufficient cardiac lymphatic drainage caused by injury, such as during surgery or myocardial infarction, results in myocardial edema (71) and increased inflammation (72) that, in turn, impair cardiac lymphangiogenesis, further contributing to pathological changes (73–75). In contrast, stimulation of lymphangiogenesis after cardiac injury increases cardiac function and is associated with decreased inflammation and myocardial extracellular volume (76, 77). A recent study highlighted the involvement of the LEC-derived paracrine signal reelin in cardiac repair (78), which is best known for its role in neuronal development and has, consequently, stimulated the field to reconsider the functional role of lymphatics in pathological conditions. Although stimulation of cardiac lymphangiogenesis appears to offer protective benefits following injury, a perplexing finding by several groups involves the loss or absence of cardiac lymphatics, which, unexpectedly, does not impair basal heart function (79, 80).

Lymphatics in the adventitial arterial wall were revealed more than a century ago (81) and identified in atherosclerotic arteries over the last 30 years (82–84). Recent studies suggest that lymphatics are critical for lipoprotein-cholesterol removal from atherosclerotic plaques (85), and stimulating lymphatic drainage in mouse atherosclerotic arteries induces plaque regression (17, 86). Additionally, impaired lymphatic function promoted inflammation, whereas prevention of lymphatic growth by blocking of VEGFR signaling aggravated atherosclerotic plaque formation (78, 87). Taken together, studies on cardiac and aortic lymphatics suggest that manipulation of the LS may improve cardiac function, decrease atherosclerosis, and improve outcomes following cardiac injury.

### Renal lymphatic system dysfunction.

Renal lymphatics are critical for kidney development, immune responses, regulation of tissue fluid and fluid balance, and the progression and persistence of renal disease (88–92). Lymphatic capillaries are sparse in the adult kidney, being located close to the glomeruli and tubules in the renal cortex. In healthy kidneys, classical lymphatic vessels are absent in the renal medulla. Renal lymphangiogenesis occurs in kidney diseases such as polycystic kidney disease (PKD), transplant rejection, acute kidney injury, diabetic nephropathy, and IgA nephropathy (92), and is correlated with renal fibrosis in chronic kidney disease (93–95). Despite a clear association between renal lymphangiogenesis and several chronic kidney diseases, varying consequences of lymphangiogenesis have been reported. For example, increased renal function may promote contradictory effects; removal of waste products and inflammatory cells benefits the kidney, while increasing inflammatory responses, via the transportation of antigen-presenting cells to hilar lymph nodes, harms the kidney (95). Differences in disease outcomes may arise from changes in lymphatic function rather than simply the expansion of nonfunctional lymphatics (92). For example, renal lymphangiogenesis is protective in mouse models of PKD and may improve blood pressure regulation (90, 96). Treatment with recombinant VEGFC in mouse models of PKD increased lymphangiogenesis and promoted clearance of pericystic inflammatory cells with resultant decreased cystic disease (97). Similarly, injection of VEGFC in wild-type mice and studies with transgenic mice overexpressing VEGFD in the tubules demonstrated increased natriuresis and decreased inflammation, kidney fibrosis, and hypertensive responses (98, 99). Further supporting the idea that improved lymphatic function decreases inflammatory responses and preserves organ function is the finding that increased lymphatic density in renal transplant biopsies or overexpression of VEGFC in mouse models of kidney transplant is associated with improved allograft function and transplant success (100, 101).

### Gastrointestinal and liver lymphatics: contribution to metabolic diseases.

The gastrointestinal (GI) tract carries out the critical function of nutrient absorption and transport. At the same time, the GI is home to a vast microbiome that might threaten the body with microbial invasion were it not for a critical checks and balances system that, in states of health, leads to a cooperative, beneficial relationship between the human body and the microorganisms of the intestinal tract. This cooperativity provides critical benefits to human metabolism, immunity, and overall physiology. Lymphatic capillaries drain the intestinal wall along the GI tract, and this lymph is then transported through collecting lymphatic vessels that lie just outside the intestine in the mesentery. Mesenteric collecting lymphatics drain to a series of intestine-draining lymph nodes, with the efferent lymph from these lymph nodes making its way to the thoracic duct.

The GI tract drains cargo to distinct lymph nodes along the different regions of the intestine (102), allowing for distinct immune responses in each region. The lymph nodes that drain the duodenum, the most proximal segment of the small intestine, are predisposed to tolerance and immunosuppression. Diseases arising from impaired food tolerance, including celiac disease associated with antigluten immunity and food allergies in general, are associated with altered responses in lymph nodes of the proximal small intestine and associated draining lymphatics. In mice, one condition that disrupts the immunosuppressive state of the proximal intestinal LS is helminth infection (102).

The blind-ended lymphatic capillaries in intestinal villi are called lacteals. They have greater surface area due to increased height of the villi in the duodenum than in other parts of the small intestine. Owing to their button-like junctions, these lymphatics efficiently pick up enterocyte-secreted, chylomicron-type lipoproteins bearing long-chain fatty acids and cholesterol esters from the diet. Closure of the button-like junctions or failure of lacteals to develop sufficient height results in impaired lipid absorption (103–105). Conversely, leak of chylomicrons from the mesenteric collecting vessels drives mesenteric or visceral adipose tissue expansion and may contribute to obesity or its complications (105–107).

The lymphatics that drain the ileum have shorter lacteals, and, contrasting with the tolerance features of the proximal LS, ileal lymph nodes are more predisposed to promoting immunity, positioning them to develop effective responses against invading organisms, as the ileum contains a denser microbial community (102). The intestinal and associated mesenteric lymphatic vessels that reach ileal-draining lymph nodes are often altered in inflammatory bowel disease (IBD). In particular, in at least some IBD patients tertiary lymphoid tissue aggregates form along the lymphatics (108) that can promote leakage of lymph while also obstructing outflow (109). The aggregates form at lymphatic valve sites in response to disease-driving cytokines that suppress critical valve-specifying genes (109). While these tertiary lymphoid structures disrupt immune cell trafficking, they may prevent dissemination of microbial signals to distal systemic environments. Interfering with valve maintenance allows for inflammatory, microbe-derived signals to disseminate beyond the intestine (110). The leakage of lymph in IBD appears to be associated with adipose expansion of the adjacent mesentery, a phenomenon called creeping fat that results in deposition of disease-associated microbes (111).

Compared with the small intestine, far less is known about colon-draining lymphatic vessels. New studies implicate small- and large-intestinal lymphatic vessels in the production of R-spondin3 that maintains stem cell niches in intestinal crypts (112–114). This secretory function for lymphatics appears to be as relevant to the small and large intestine as to the cardiac system (74), but more studies are needed.

Rather than entering the lymphatic vasculature, the majority of molecular cargo absorbed and released from enterocytes is smaller than chylomicrons and thus freely enters the fenestrated venous vasculature that overlays the lacteals (115). Venous outflow from the intestine flows into the portal vein that serves as the major blood supply for the liver. Thus, chylomicrons that carry fats are the exceptional nutrient type that routes through intestinal lymphatics, while the liver receives and serves to filter and metabolize much of the molecular cargo crossing the intestinal wall. This feature not only allows the liver access to intestinal cargo for metabolic purposes, but also positions the liver to protect the central blood supply from toxic or inflammatory exposure, thereby reducing the probability of acute events that have the potential to escalate into deadly outcomes like shock, sepsis, or disseminated intravascular coagulation.

Future research is needed to understand how cargo arriving to the liver is separated for further transport out of the liver via liver-draining lymphatics versus the hepatic vein. The liver-draining lymph makes a substantial contribution to lymph return at the thoracic duct; 25% to 50% of lymph passing through the thoracic duct arises in the liver (70). Liver lymphatics are challenging to study since many markers used to identify lymphatics in other organs are not lymphatic-selective in the liver. Cirrhosis of the liver substantially increases the output of lymph from the liver; however, it is unclear whether the nature of the cargo that is routed to lymph versus venous outflow of the liver is altered in conditions like cirrhosis. Lymphatic proliferation and stability are also altered in other liver conditions like nonalcoholic and alcoholic fatty liver disease (116, 117), but much remains to be studied as to whether such alterations impact the course of disease progression in the liver.

Studies have shown that obesity can lead to impaired lymphatic function (118). This dysfunction contributes to the development of obesity-related comorbidities by promoting tissue inflammation, impairing lipid metabolism, and altering immune cell trafficking (119). Given the link between obesity and lymphatic dysfunction, targeting the LS may represent a therapeutic strategy for obesity and its associated comorbidities. Preclinical studies have demonstrated that stimulating lymphangiogenesis or enhancing lymphatic function can improve obesity-related inflammation and metabolic dysfunction (55).

As our understanding of the role of lymphatics in obesity continues to evolve, there are several objectives for researchers to meet, including (a) identifying mechanisms that regulate lymphatic vessel formation and function in response to obesity; (b) determining the role of lymphatic dysfunction in the development of obesity-related complications, such as insulin resistance and atherosclerosis; (c) investigating the crosstalk between the LS and other tissues, such as adipose tissue and the immune system, in the context of obesity; (d) exploring interventions to improve lymphatic function and ameliorate obesity-related complications; and (e) developing imaging techniques for the noninvasive assessment of lymphatic function in obesity.

### Autoimmunity and lymphatic function.

Alterations in lymphatic function have been implicated in several autoimmune diseases, including multiple sclerosis (see *CNS lymphatic dysfunction* section above), Castleman disease, dermatomyositis, systemic sclerosis, systemic lupus erythematosus, and rheumatoid arthritis (RA) (120). These observations are not surprising given the key role lymphatics play in concentrating the peripheral tissue antigen landscape into draining lymph nodes, active sites of innate and adaptive immune responses (121). LECs contribute to this process by producing chemokines such as CCL21 and CCL19 that guide positioning of dendritic cells within lymph nodes (122). LECs can also directly modulate immune cell activity by producing growth factors and cytokines including TGF-β1, IL-7, and colony-stimulating factor 1 (CSF1) (123–126). More recent studies have shown that LECs express MHCII complexes and can present peripheral tissue antigens or transfer immune complexes to other antigen-presenting cells. In contrast to professional antigen-presenting cells (127–130), antigen presentation by LECs occurs without costimulatory molecules and is thought to inhibit immune responses. In addition, LECs can present antigens together with programmed cell death ligand 1 (PD-L1) and inhibit cytotoxic T cell activation (131, 132) or mediate tolerance through an autoimmune regulator–independent (AIRE-independent) mechanism (133). Interestingly, mice that lack cutaneous lymphatics develop autoantibodies (134).

The effects of lymphatic function in RA have been the subject of intense study and have led to clinical advances that have greatly improved treatment outcomes. In RA, autoimmune responses attack the synovium, resulting in inflammation and the release of inflammatory cytokines that further damage the synovium. In the early stages of synovial inflammation, lymphangiogenesis and increased lymphatic pumping increase clearance of inflammatory cells and cellular debris, thus enabling partial resolution of the inflammatory process (135, 136). However, persistent inflammation and inflammatory cytokines damage the LECs and LMCs in the afferent lymphatics and draining lymph nodes, leading to the collapse phase of the disease (137). Impaired lymphatic function prompts accumulation of fluid in the joint, and lymph node sinuses become obstructed by B cells (138). These changes lead to increased joint inflammation, synovial hyperplasia, and joint destruction (139). Immune modulating treatments for RA such as anti-TNF or anti-CD20 antibodies not only decrease inflammation but also improve lymphatic function (137, 140).

### Lymphedema.

Lymphedema is a chronic disease characterized by impaired lymphatic function causing tissue swelling in the skin. Impaired lymphatic function results in accumulation of ISF, adipose deposition, and soft tissue fibrosis. Patients with lymphedema develop progressive swelling and heaviness of the affected limbs. Lymphedema can be broadly categorized as primary lymphedema resulting from congenital abnormalities of the LS or secondary lymphedema resulting from trauma, infection, or injury to the LS.

The incidence of primary lymphedema is not well understood; however, the disease is estimated to occur in 1 of every 6,000 people (141, 142). Congenital abnormalities of the LS arise from spontaneous or inherited mutations of *VEGFR3* or other genes. Primary lymphedema most commonly presents in adolescence or later in life and, for unknown reasons, affects females more frequently than males.

Secondary lymphedema is the most frequent cause of lymphedema worldwide. In developing countries, filariasis, a parasitic infection, is the most common cause of secondary lymphedema. Estimates suggest that over 100 million patients suffer from filariasis (143). In Western countries, the most common cause of lymphedema is surgical injury associated with cancer treatment. The disease may also develop secondary to trauma, radiation, obesity, or other external factors. It is estimated that 1 in 1,000 patients suffers from secondary lymphedema, but the true incidence may be higher (144). Breast cancer treatment is the most common cause of secondary lymphedema in Western countries because of the high prevalence of this cancer; however, lymphedema also occurs commonly after treatment of other solid tumors (145), with varying incidence depending on the cancer type, diagnostic methods, length of follow-up, and anatomic variabilities (146–148).

The pathophysiology of secondary lymphedema is complex and involves inflammation, fibrosis, impairment of collateral lymphatic formation, valvular failure, decreased lymphatic pumping, and lymphatic leakiness (144). Secondary lymphedema typically develops in a delayed fashion after the initial surgical insult, suggesting that lymphatic injury is the initiating factor. ISF accumulation is thought to promote development of chronic inflammation, leukotriene activation, and adipose deposition (149–154). T helper cell infiltration and differentiation along the T helper 2 (Th2) lineage is an important regulator of disease development, and experiments using animal models show that Th2 cells are necessary and sufficient for the development of secondary lymphedema (155–157). Recent clinical trials, aligning with studies in animals, show promise for antiinflammatory treatments (158, 159). Development of effective therapies is an active area of research and an important unmet need.

### Lymphatic anomalies.

Anomalies of the LS can cause substantial morbidity and in some cases mortality. Complex lymphatic anomalies are rare, sporadically occurring diseases characterized by multifocal lymphatic malformations (160). Kaposiform lymphangiomatosis (KLA) is a potentially life-threatening lymphatic anomaly characterized by diffusely abnormal lymphatics in multiple organs (161). The etiology of KLA is not well understood but involves a somatic activating mutation on the *NRAS* oncogene (Q61R) in some patients with KLA (162, 163). This mutation leads to the activation of MAPK and PI3K signaling pathways, resulting in unchecked cell proliferation. Recent studies have identified elevated serum levels of angiopoietin-2 expression as a biomarker of the disease that is useful for following disease progression (164). Therapies have likely improved the prognosis (165); however, more studies are needed to quantify the prevalence of KLA and to develop more specific treatments. Gorham-Stout disease (GSD) is another rare disease characterized by progressive bone loss and lymphatic vessel abnormalities of the bone and other organ systems (165–167). The etiology of GSD is incompletely understood but is thought to involve abnormal growth and invasion of lymphatic vessels into the bones leading to bone loss, pathological fractures, and bone deformity. VEGFC and VEGFD are the most important factors that drive lymphatic vessel formation from preexisting vessels (168, 169). These factors might account for the effects of GSD, as uncontrolled VEGFC expression induces lymphatic invasion of bone and osteolysis (170, 171). An activating somatic mutation in the *KRAS* proto-oncogene (G12V) was identified in a patient with GSD (172), and a mouse model was developed for GSD that revealed lymphatic developmental defects with fewer lymphatic valves (172).

A better understanding of cellular mechanisms regulating abnormal lymphatic development such as hyperactive RAS/MAPK signaling or abnormalities in PI3K/AKT pathways may lead to treatments for rare diseases that cause notable morbidity and mortality. An example can be found in the successful treatment of lymphatic anomalies in a patient with Noonan syndrome, a RASopathy affecting multiple organ systems. DCMRL revealed severe visceral lymphatic flow derangements that were linked to gastrointestinal bleeding and protein-losing enteropathy (PLE). Treatment with a MEK inhibitor to target RAS signaling resolved the lymphatic defects and led to dramatic improvement in clinical outcomes and quality of life (173).

Central lymphatic flow defects can lead to PLE or plastic bronchitis, and both complications are linked to aberrant lymphatic patterning and flow (174). These complications have been directly connected to unusual lymphatic patterning that may bypass or be linked to the thoracic duct. Both are also linked to lymph backflow into the aberrant lymphatic structures. For PLE, backward-flowing lymph pushes protein and immune cells into the lumen of the intestine, causing hypoalbuminemia and low T cell counts (174). Alternatively, a fistula may develop between visceral lymphatics and the duodenum, given the convergence of many vascular and lymphatic structures in the visceral space where the pancreas, liver, and duodenum come into proximity with each other (175). Embolization that ablates access of lymph to the aberrant lymphatic vessels can effectively treat some patients (174).

## Lymphatic imaging and mapping

Lymphatic imaging is critical for diagnosing and treating lymphatic conditions. To this end, several lymphatic disorders have key imaging features such as lymphatic malformations, structural anomalies, and chylous effusions (176). For decades the majority of clinical imaging was performed using two techniques: pedal lymphangiography and lymphoscintigraphy (177). However, the last 10 to 15 years has seen an increase in the number of lymphatic mapping techniques (146), reflected in the relatively small but growing number of publications on lymphatic imaging (178). Innovative technologies with improved resolution and enhanced sensitivity have led to the discovery of lymphatic variants, further revealing the importance of lymphatic anatomy to the pathophysiology of disease (Supplemental Table 1). These technologies include intranodal lymphangiography, DCMRL, liver lymphangiography, mesenteric lymphangiography, sodium MRI, and computed tomography lymphangiography. A remaining challenge in the field is the establishment of intraoperative imaging methods. A potential strategy is to use indocyanine green (ICG) lymphography, in which near-infrared cameras detect intradermally injected ICG (179, 180). It is important to note that this procedure uses ICG as an off-label drug, though it is FDA approved for intravenous injections. ICG lymphography also has some limitations, including relatively low depth of penetration below the skin and lack of quantitative measures that can follow clinical changes longitudinally. The strength and limitations of clinical imaging methods have been the focus of other NIH-sponsored workshops (2, 146), have been reviewed elsewhere (176–178), and were discussed during the “Yet to be Charted: Lymphatic System in Health and Disease” workshop (1).

## Research opportunities and challenges

The last decade has seen remarkable progress in the field of lymphatic research. These changes include recent efforts to map the human LS by the Human BioMolecular Atlas Program (HuBMAP) (181) and the establishment of two NIH categories by the Research, Condition, and Disease Categorization (RCDC) system (182) (“lymphatic research” and “lymphedema”), which enable stakeholders to track NIH funding and research progress on lymphatics. Furthermore, a recent portfolio analysis of NIH-funded research with the term “lymphatic research” showed 11 NIH institutes funding lymphatic research projects; the success rate of these grant applications was comparable to the overall success rate of all NIH grant applications and has increased over the last five years (Figure 2, D–F).

Despite these achievements, workshop participants identified many research challenges and opportunities for the field, including the development of animal models and methods to study lymphatic cell function, contractility, and valvular function. Additional priorities include targeted treatments to modulate lymphatic function, profiling of lymphatic cell heterogeneity, and characterization of the role of the lymphatic vasculature in immune activation.

Many transgenic mouse models have been developed to study the LS (Supplemental Table 2). Lymphatic-specific Cre drivers (e.g., *Prox1*, *Vegfr3*, *Lyve1*) are now widely available. These models allow targeted manipulation of the lymphatics, but the results must be interpreted with care because these markers are expressed by other cell types. Strain-specific issues may also influence mouse models and complicate the translation of experimental findings. The study of LMCs and the regulation of lymphatic contractile and valvular function has been particularly difficult. One common approach uses optical imaging with ICG or other fluorescent dyes; however, quantification of lymphatic dynamics (e.g., stroke volume, ejection fraction, pumping frequency) is not easily accomplished. Other strategies rely on the analysis of isolated lymphatic vessels in vitro; however, this approach is technically challenging and not widely available. Thus, the recent development of LMC-specific Cre drivers addresses an important need that requires additional study (53, 183). Large-animal models of lymphatic dysfunction are also needed to better represent the clinical scenario. CRISPR/Cas9 in particular has the potential to provide a means of producing lymphatic-specific large-animal models.

Targeted modulation of lymphatic function to increase lymphatic contractility, decrease lymphatic leakiness, or otherwise improve lymphatic transport capacity was identified as another area of therapeutic opportunity. Conventionally, supraphysiological doses of lymphangiogenic growth factors such as VEGFC or injection of VEGFR3 neutralizing antibodies have been used. However, these experimental manipulations have off-target effects on other cells (e.g., blood endothelial cells and macrophages). Thus, treatments with low off-target effects are needed to move the field forward and clinically translate exciting research findings.

Profiling LEC and LMC heterogeneity at single-cell resolution using advanced omics and spatial mapping techniques represents another growth opportunity. Notable metabolic and immunological differences have been identified in LECs and LMCs across various organ systems. Ambitious efforts to create reference atlases of healthy and diseased tissues by the NIH HuBMAP, the Human Cell Atlas Consortium, and others have the potential to reveal unique biological insights, discover cell types, accelerate drug discovery, and establish promising therapeutic interventions for human diseases (184).

## Clinical challenges and opportunities

The workshop deliberations included clinical researchers, patients, and advocates. Stakeholders identified multiple unmet needs related to patient care, including epidemiological studies that define the prevalence of lymphatic diseases, risk prediction algorithms to stratify patients, lymphatic centers of excellence for both adults and children, physician education and awareness of lymphatic diseases, and advanced strategies for the prevention and treatment of lymphedema. Patients raised the audience’s awareness of the psychosocial effects of lymphatic disorders beyond depression and infertility. Attendees discussed sex as a biological variable in lymphatic diseases, as well as racial and socioeconomic factors that contribute to disease severity and access to care.

Surgical treatments for lymphedema have had a resurgence in the past 10 years due to advances in our understanding of lymphedema pathophysiology and microsurgical techniques. Lymphovenous bypass, a procedure in which leaking lymphatics are connected to a nearby vein, thus bypassing and improving lymphatic drainage, has shown symptomatic relief and decreased pathological changes of lymphedema (185). Vascularized lymph node transplantation, a procedure in which lymph nodes from a healthy donor site are transplanted to the affected extremity, has also been effective in carefully selected patients (186, 187). However, large-scale prospective trials are needed to optimize surgical algorithms, study the long-term outcomes, and improve health insurance coverage for these procedures. It is also likely that a combination of surgery and pharmacological treatments will be more successful for treating lymphedema (144).

Functional, noninvasive, quantitative measures are needed to analyze and quantify lymphatic function. These methods will facilitate rigorous analysis of surgical or medical intervention outcomes, providing an important advancement over current approaches that rely on secondary changes such as fluid accumulation or fibroadipose deposition. Improved imaging modalities to study the complexity of the LS, lymphatic valve function, and lymphatic anatomy are also needed. Identifying biomarkers of lymphatic function would also be a major clinical advance.

As with research studies using animal models, characterization of LEC and LMC heterogeneity at single-cell resolution in large numbers of individuals is key to determining the effects of comorbid conditions on gene expression and function. It is likely that demographic features such as race or comorbid conditions such as obesity, age, chemotherapy, radiation therapy, and other factors substantially modify LEC and LMC gene expression; and understanding these changes can identify areas that require additional study and treatment options (188).

## Conclusions

The field of lymphatic research has a captivating history and a promising future. Our knowledge of the LS and its importance in health and disease has grown substantially over the last two decades. In recent years, interest in lymphatic disorders has increased, with many enthusiastic young investigators and physician-scientists now engaged in lymphatic research. There is potential for new cross-disciplinary collaborations that may identify novel, targeted therapies. The NIH, the largest public funder of biomedical research in the world, continues to invest in basic and translational research to advance the last frontiers of medical research. The ongoing challenge to develop and disseminate intervention strategies that enhance the quality of life of those living with lymphatic disorders now provides opportunities for early-career investigators.

## Author contributions

BJM and AJR are co–first authors. Their names appear in alphabetical order.

## Supplementary Material

Supplemental data

## Version 1 09/01/2023

Electronic publication
