# Supplementary material for: The emerging importance of lymphatics in health and disease: an NIH workshop report
Source: J Clin Invest. 2023 Sep 1;133(17):e171582. doi: 10.1172/JCI171582 (PMC10471172; doi:10.1172/JCI171582)
Supplement: Supplemental data [file jci-133-171582-s159.pdf]

Date	Events and discoveries that advanced lymphatic research
1600 B.C.E.	Lymph nodes first described in Egyptian hieroglyphs (the Edwin Smith Medical Papyrus) (1).
400 B.C.E.	Lymph nodes in the mesentery, axilla, and other areas of the body reported in the Hippocratic Corpus (2-6).
460-377 B.C.E.	Hippocrates defines the terms white blood and chyle (2, 7).
384-322 B.C.E.	First (assumed) reports of lymphatic vessels by Aristotle (3, 5, 6, 8, 9).
335-280 B.C.E.	Herophilus identifies milky veins (lacteals) and mesenteric lymph nodes (7-10).
310-250 B.C.E.	Erasistratus of Chios describes chyle and first description of mesenteric lymphatic vessels (7-9).
70-110	Rufus of Ephesus credited with first describing the thymus gland (11).
129-199	Galen of Pergamum describes the mesenteric lymph nodes and lacteal vessels containing chyle (11).
607-690	Paul of Aegina describes the tonsils (11).
1514-1564	Andreas Vesalius advances the field of human anatomy (3, 12).
1520-1574	Bartolomeo Eustachius credited with initial description of thoracic duct in horses (3, 8, 10, 13).
1536	Niccolo Massa is attributed with describing the renal lymphatic system (3, 11, 14).
1622	Lacteals discovered by Gaspare Aselli (5, 7, 9, 15).
1629	Jacques Mentel reports that mesenteric lacteals end in thoracic duct before entering bloodstream (7, 9).
1647-1650s	Jean Pecquet discovers the cisterna chyli, thoracic duct, and presence of valves within lymphatics (7, 9).
1651	Discovery of the thoracic duct and entry into the veins by Jean Pecquet (16, 17).
1650s	Discovery and description of the human lymphatic system by Olaus Rudbeck and Thomas Bartholin (16, 18, 19). Francis Glisson (1597-1677) credited with deciphering the anatomo-physiology of lymphatics with his pupil George Joyliffe. George Joyliffe (1621-1658) contributed with description of lymphatics, distinguishing between lacteals and non-lacteal vessels (4, 20).
1666	Marcello Malpighi describes the two-compartment structure and function of the spleen in *De Viscerum Structure* (21). Malpighi also pioneered the application of microscopy to medicine.
1701	Frederick Ruysch describes the morphology and function of lymphatic valves using innovative preservation techniques (22).
1745	Johann Nathanael Lieberkühn identifies the origin of lacteals in the intestines (11, 20).
1757	Spread of cancer along the lymphatic system first reported by Henri Francois LeDran (11, 23).
1780s	Foundation of modern knowledge on the function of lymphatics established by Hunter brothers John and Williams, William Hewson, and William C. Cruikshank (16, 17).
1787	Giovanni Paolo Mascagni creates the first comprehensive lymphatic atlas, mapping 50% of all lymphatic vessels of the human body (24).
1856	Rudolf Virchow is the first to describe the involvement of lymph nodes in cancer including leukemia, lymphoma, and pseudoleukemia. Virchow’s early work provided a valuable framework for sentinel node biopsy by presenting the hypothesis that lymph nodes act as a barrier to tumor dissemination (23).
1858	Carl Friedrich Wilhelm Ludwig proposes the theory of lymph formation by filtration from blood capillaries (8, 25).
1874	Marie Philibert Constant Sappey creates a detailed atlas of the cutaneous lymphatic system and deeper lymphatic trunks (26).
1892	Primary lymphedema classified as Milroy disease (27).
1892-1896	Seminal experiments by Ernest Starling establish the Starling principle and the regulation of interstitial fluid (28).
1898	Primary lymphedema classified as Meige disease (29).
1897-1913	Ranvier, Sabin, and Lewis conclude that lymphatics emerge from the venous system (7, 30).
1902-1904	Sabin concludes that lymphatics originate from veins; most widely accepted view of lymphatic development was proposed by Sabin in the early twentieth century (7, 30).
1911	Sabin describes lymphatics as a closed system composed of veins lined by an endothelium (16, 31).
1923	Braithwaite reports lymphatic drainage from abdominal cavity to “glands sentinel”, providing further support for the sentinel node theory (11, 32).
1935-1942	Cecil Drinker profiles the composition of lymph from the lungs and the heart, demonstrating a critical role for the lymphatic system in the absorption of protein from interstitial tissues (8, 33, 34).

Date	Events and discoveries that advanced lymphatic research
1938	Henri Rouvière publishes textbook “Anatomy of the Human Lymphatic System”, still regarded as the most comprehensive treatise on lymphatic anatomy (35).
1939	Paul Patek performs a detailed assessment of the morphology and anatomy of cardiac lymphatics in a wide range of mammals (36)
1940s	Use of radioactive iodine to demonstrate that lymph originates from the blood stream (16).
1949	First documented study in PubMed describing the presence of intrinsic contractility in human lymphatics (37). Original description of pumping activity of lymphatic vessels described by Arnold Heller in 1869 (38).
1950s	Lymphoscintigraphy is first established in the 1950s and is still regarded as the “gold-standard” for lymphedema diagnosis (39).
1952	Kinmonth achieves the first good lymphangiogram through injection of diodone, a water-soluble radio-paque contrast medium, in humans (40).
1958-1965	Leonetto Comparini and colleagues make several significant contributions related to the molecular and morphological characterization of lymphatic capillaries, pre-collecting lymphatic vessels, and collecting lymphatic vessels. He additionally contributed reconstructions of the hepatic lymphatic system (3, 41).
1960	Ernest Gould is the first to use the term “sentinel node” in his pathology report of “lymph node with metastatic tumor” (42).
1961	Jacques Miller determines the function of the thymus gland, the last organ of the human body to have its mechanisms fully understood (43).
1964	Directed mechanism of lymphocyte migration/homing into lymph nodes described by Gowans and Knight (44).
1975-1980	Experiments in embryos confirm origin of lymphatics from venous endothelium (7).
1992-2001	Kari Kustaa Alitalo and colleagues elucidate the growth factor/receptor system governing lymphatic vessel formation and lymphatic metastasis of tumors (45-51).
1996-1998	VEGFR3 shown to be required for angiogenesis and lymphangiogenesis; One of the first lymphatic markers to be identified (7, 47, 52) Identification of LEC-specific markers leads to development of transgenic animal models (Supplemental Table 2).
1997	Group of Irving Weissman define lymphoid tissue inducer cells, a subset of innate lymphocytes that are essential for the formation of secondary lymphoid organs during embryogenesis (53).
1999	LYVE-1 established as a LEC marker (7, 54).
1999	*Prox1* expression shown to be critical for lymphatic vascular development (55).
1999	Podoplanin is established as a valid marker for lymphatic endothelia using immunohistochemistry and specific antibodies (56).
2001	Demonstration that malignant tumors can directly activate lymphangiogenesis and lymphatic metastasis (49, 50, 57, 58).
2002	TNLCC (Trans-NIH Lymphatic Coordinating Committee) established to coordinate trans-NIH activities on lymphatic biology and diseases.
2003	Identification of podoplanin as a marker for differentiated LECs (7).
2003	Early lymphatic mapping in animal models using indirect computed tomography lymphography (59).
2004	VEGFC, best-characterized VEGFR3 ligand, is required for LEC budding but not commitment (7, 60).
2004	Gordon Research Conference hosts the first dedicated conference on lymphatics titled, “Molecular Mechanisms in Lymphatic Function and Disease.” (https://www.grc.org/molecular-mechanisms-in-lymphatic-function-and-disease-conference/2004/).
2007	Lineage tracing experiments in Cre/loxP transgenic mice confirm that majority of embryonic mammalian LECs are venous derived (61).
2007	Indocyanine green lymphangiography first used for lymphedema evaluation (62, 63).
2008	Transcription factor *Sox18* shown to be important for Prox1 induction (64).
2009	NIH Research Festival 2009. Symposium Lymphatic Biology and Disease: the Cinderella of the Vascular System Finally Gets Invited to the Ball (65).
2012	Nadolski and Itkin demonstrate the use of ultrasound-guided intranodal lymphangiogram for thoracic duct embolization in a feasibility study (66).
2012-2017	The glymphatic system is first described in mice (67) and then demonstrated in humans (68). The meningeal lymphatic network is described in mice (69, 70) in 2015 and humans (71) in 2017.
2014-Present	Evidence of a non-venous origin for a small percentage of tissue-specific LECs (7, 72-74).
2014-2016	Dynamic contrast enhanced magnetic resonance (MR) lymphangiography is first established in swine (75) and expanded to the clinic by Itkin, Dori, and colleagues (76-78).

Date	Events and discoveries that advanced lymphatic research
2014	Schlemm's canal of eye reveal lymph-like qualities (7, 79-82).
2015	Workshop: The Third Circulation: Lymphatics as Regulators in Health and Disease (83).
2016	The Human Cell Atlas, an international effort to characterize every cell in the human body at single cell resolution using advanced technologies, is established (84).
2018	Major trans-NIH effort, the Human BioMolecular Atlas Program (HuBMAP), supported by the NIH Common Fund, brings together biologists, pathologists, and data scientists to create an open and global platform to map the cells of all the major human organ systems, including the lymphatic system (85, 86).
2018	The clinical application of intranodal mesenteric lymphangiography for managing postoperative recalcitrant chylous ascites is evaluated in a feasibility study (87).
2021	Workshop: Yet to Be Charted: Mapping the Lymphatic System Across Body Scales and Expertise Domains (88).
2021	Activating somatic mutation in KRAS was identified in a patient with Gorham-Stout disease (GSD). A GSD mouse model was developed revealing lymphatic developmental defects with significantly fewer lymphatic valves (89).
2022	Workshop: Yet to Be Charted: Lymphatic System in Health and Disease (90).
2022	New Research, Condition, and Disease Categorization (RCDC) terms established, "Lymphatic Research" and "Lymphedema" (91).
2023-Present	Ongoing efforts to establish a National Commission on Lymphatic Diseases after Congressional Mandate on FY2021 appropriations for the Department of Health and Human Services (92).

Supplemental Table 1. A comprehensive timeline of lymphatic discoveries and NIH-led activities and funding opportunities.

The field of lymphatic research spans several millennia, reflecting a timeless desire to understand the system and its parts. Summary of landmark findings, international workshops, and NIH-led activities is provided with key references for further review. For a more extensive account of the scholars, discoveries, and priority disputes we refer the reader to several excellent reviews on this topic (3-5, 7, 8, 11, 16, 20).

Abbreviations: B.C.E.-Before Christian Era

Marker	Mouse strain	Description and/or application(s)	Year
ANG2	Angpt2^{tm1Gdy}	Angiopoietin 2 knockout mice created by replacing coding portion of Angiopoietin 2 with *lacZ* gene encoding galactosidase. Gross abnormalities of the lymphatic system observed, providing a model for studying lymphatic insufficiency (93).	2002
Claudin 11	Cldn11-CreER^{T2}	Transgenic mouse expressing tamoxifen inducible CreERT2 under control of the *cld11* (Claudin 11) promoter. Allows for functional studies of lymphatic valves in a selective and temporally controlled manner (94).	2021
FOXC2	Foxc2^{tm1Miu}	*Foxc2* heterozygote mice recapitulate the lymphatic and ocular phenotype of lymphedema-distichiasis syndrome (95).	2003
LYVE1	B6.129S1-Lyve1$^{tm1Lhua/J}$	Knockout mice display functional alterations of lymphatic capillary vessels and increased interstitial-lymphatic flow (96).	2006
LYVE1	Lyve1^{tm1Dgjk}	LYVE1 deficient mice created through targeted replacement of *Lyve1* with *TM-lacZ* reporter gene. Lymphatic networks, lymph nodes, and immune cell trafficking are normal in these mice (97).	2007
LYVE1	Lyve1CreERT2	Inducible Cre recombinase-estrogen receptor construct targeting lymphatic epithelial cells (LECs). This transgenic mouse allows for visualization and selective deletion of LEC-specific genes in entire lymphatic vessels or LEC progenitors depending on the dosing schedule of 4-hydroxytamoxifen and genetic cross to floxed mice (98).	2016
Podoplanin	Pdpn-GFPCre	Transgenic mouse expressing Cre under control of a 5' upstream regulatory region of the *Pdpn* gene, providing a better tool to visualize lymphatics and their function in mice (99).	2018
Prox1	Prox1/LacZ	LacZ insertion into *Prox1* allows for selective and temporal expression of *Prox1 (100)*.	1999
Prox1	prox1-mOrange2-pA-BAC	*Prox1* promoter driven expression of fluorescent protein mOrange2 for intravital visualization of lymphatic growth and function in fetal and adult mice (101).	2011
Prox1	Prox1-CreERT2	Transgenic line expressing tamoxifen-inducible Cre recombinase (*CreER^{T2}*) under control of the *Prox1* gene promoter. When crossed to transgenic mice carrying fluorescent reporter proteins or floxed genes, the resulting progeny can be used to visualize lymphatics and/or perform functional studies on conditional knockouts, respectively (102).	2011
Prox1	BACTg(Prox1-EGFP)	Transgenic mouse expressing Green Fluorescent Protein (GFP) in lymphatic but not blood vascular endothelial cells using a bacterial artificial chromosome (BAC) approach (103).	2011
Prox1	ProxTom	Contains all *Prox1* regulatory sequences for expression of RFP tdTomato in lymphatic vessels. Demonstrates intense expression in endogenous lymphatic vessels (104).	2012
Prox1	Prox1-Cre-tdTomato	tdTomato reporter mouse crossed with line expressing Cre recombinase under control of the *Prox1* promoter (Prox1-Cre-ERT2 line (102) allows for intravital microscopy of dendritic cell migration into and within lymphatic vessels and fluorescence-activated single cell analysis of lymphatic endothelial cells (105).	2015
Prox1	Prox1-GFP/Flk1::myr-mCherry mice	Dual reporter system where lymphatic vessels emit green and blood vessels emit red fluorescence, allowing for simultaneous observation of lymphangiogenesis and angiogenesis in vivo (106).	2015
Prox1	Prox1-GFP	Prox1-GFP mice on the wild-type C57BL/6 background. Applications include intravital imaging of newly formed lymphatic vessels and valves in the cornea to observe lymphangiogenesis stimulated by injury and/or therapeutic intervention (107).	2016
Prox1	Prox1-tdTomato	Strong tdTomato expression under control of *Prox1* for evaluation of lymphangiogenesis and lymphatic differentiation (108).	2016

Marker	Mouse strain	Description and/or application(s)	Year
Prox1	Prox1-EGFP BAC	Transgenic lymphatic reporter rat expressing EGFP via BAC with mouse *Prox1* regulatory elements. Allows for visualization of all lymphatic vessels including the lymphatics of the central nervous system and Schlemm's canal (109).	2017
Prox1	Prox1CreERT2 Foxo1$^{flox/flox}$	Use of tamoxifen-inducible LEC-specific Cre line (Prox1Cre ERT2) crossed to *Foxo1*$^{flox/flox}$ mice to evaluate the role of *Foxo1* on lymphatic valve function in a temporally controlled manner. Deletion of *Foxo1* in these mice activated the lymphatic valve formation process.	2021
VEGFC	Tg(KRT14-VEGFC)1Ali	Overexpression of VEGF-C in skin resulting in hyperplasia of lymphatic vasculature (110).	1997
VEGFC	VEGFC/LacZ	LacZ reporter gene placed in exon of *Vegfc* to create postnatal lethal *Vegfc* knockout mouse. VEGFC was shown to be dispensable for LEC lineage commitment but critical for sprouting proceeding lymphatic endothelial specification in embryonic mice (60).	2004
VEGFD	VEGFD/LacZ	LacZ reporter in *Vegfd* exon generates *Vegfd* knockout mice which showed no evidence of altered interstitial fluid volume, chyle accumulation, or swollen tissue (111).	2005
VEGFR2	Lyve-1$^{(wt/Cre)}$;Vegfr2$^{(flox/flox)}$	Mice develop functional lymphatic vasculature in the absence of VEGFR2 but exhibit lymphatic hypoplasia (112).	2013
VEGFR3	Vegfr3^{lacZ}	lacZ gene under transcriptional regulation of *Vegfr3* allows for targeted editing of the *Vegfr3* locus in embryonic mice with staining revealing typical lymphatic development (52, 113).	1998, 2001
VEGFR3	C3H101H-Flt4Chy/H	Inactivating *Vegfr3* mutation characterized by chylous ascites and swelling of limbs due to a lack of cutaneous but not visceral lymphatic vessels. Observed phenotype models Milroy's disease in humans (114).	2001
VEGFR3	Tg(KRT14-FLT4/IGHG1)#Ali	Soluble VEGFR-3 fusion protein recapitulates several symptoms of human lymphedema including dermal fibrosis in aged mice and thickening of the dermis and subcutaneous fat layer. Transgenic mice lack cutaneous lymphatic vessels (51).	2001
VEGFR3	VEGFR3-YFP	BAC *Vegfr3:YFP* transgenic line expressing Yellow Fluorescent Protein (YFP) under the control of *Vegfr3* regulatory sequences (115).	2011
VEGFR3	Vegfr3(EGFPLuc)	EGFP-luciferase fusion protein expressed under control of *Vegfr3*. Bioluminescence imaging using luciferase allows tracking of tumor-induced lymphangiogenesis at the tumor periphery and in lymph nodes (116).	2012
VEGFR3	Vegfr3-tdTomato	Flt4 regulated expression of tdTomato allows for visualization of lymphatic vessels in the lung, kidney, heart, diaphragm, intestine, mesentery, liver, dermis and other organs by confocal laser scanning, light sheet fluorescence, and two-photon microscopy in adult and embryonic mice (117).	2021

Supplemental Table 2. Overview of transgenic animals used for lymphatic research. Genetically engineered mice expressing fluorescent reporter proteins or Cre recombinase under control of LEC-specific promoters such as *Lyve1, Prox1, Vegfr3*, and *Pdpn* have been widely used to study the lymphatic system. Here, we summarize of few of these strains and provide original references for further review. A limitation of these models is the expression of LEC-specific markers by other cell types and tissues, e.g., *Lyve1* by macrophages, *Prox1* by cardiomyocytes, and *Pdpn* (Podoplanin) in the injured heart (118-120). Additional genetic strains can be found using the Mouse Genome Database (MGD; http://www.informatics.jax.org) (121).

References

1. Breasted JH. *The Edwin Smith Surgical Papyrus V1/2: Facsimile Plates And Line For Line Hieroglyphic Transliteration.* Kessinger Publishing, LLC; 2010.
2. Potter P. *Hippocrates, volume VIII. On Glands.* Cambridge, MA: Harvard University Press; 1995.
3. van Schaik CJ, et al. The lymphatic system throughout history: From hieroglyphic translations to state of the art radiological techniques. *Clin Anat.* 2022;35(6):701-10.
4. Ambrose CT. Immunology's first priority dispute—An account of the 17th-century Rudbeck–Bartholin feud. *Cell Immunol.* 2006;242(1):1-8.
5. Kanter MA. The Lymphatic System: An Historical Perspective. *Plast Reconstr Surg.* 1987;79(1).
6. Crivellato E, and Ribatti D. A portrait of Aristotle as an anatomist: Historical article. *Clin Anat.* 2007;20(5):447-85.
7. Escobedo N, and Oliver G. Lymphangiogenesis: Origin, Specification, and Cell Fate Determination. *Annu Rev Cell Dev Biol.* 2016;32(1):677-91.
8. Leeds SE. Three centuries of history of the lymphatic system. *Surg Gynecol Obstet.* 1977;144(6):927-34.
9. Chikly B. Who Discovered the Lymphatic System? *Lymphology.* 1997;30:186-93.
10. Fulton JF. The early history of the lymphatics. *Bull Hennepin Co M Soc* 1938;9:5.
11. Loukas M, et al. The lymphatic system: a historical perspective. *Clin Anat.* 2011;24(1098-2353 (Electronic)):807-16.
12. Vesalius A. *De Humani Corporis Fabrica.* Basel: J. Oporinus; 1543.
13. Eustachi B. *Opuscula Anatomica.* Venetiis: V. Luchinus; 1564.
14. Massa N. *Liber introductorius anatomiae, siue dissectionis corporis humani.* Venezia: F. Bindoni & M. Pasini; 1536.
15. Aselli G. *De lactibus sine lacteis venis, quarto vasorum mesaraicorum genere, novo invento. Dissertatis qua sententiae anatomicae multae uel parum perceptae illustratur.* Milan, Italy: J.B. Bidellum; 1627.
16. Mayerson HS. Three Centuries of Lymphatic History - an Outline. *Lymphology.* 1969;2:143-50.
17. Pecquet J. *Experimenta nova anatomica quibus incognitum hactenus chyli receptaculum, et ab eo per thoracem in ramos usque subclavios vasa lactea detegunter.* Paris: Seb. Cramoisey and Gab. Cramoisey; 1651.
18. Rudbeck O. *Nova Exercitatio anatomica, exhibens ductos hepaticos aquosos, et vasa glandularum serosa, nunc primum inventa, æneisque figuris delineata.Cui asccessere aliae ejusdem observationes anatomicae.* Arosiae: E. Lauringer; 1653.
19. Bartholin T. *Vasa lymphatica, nuper Hafniae in animantibus inventa et hepatis exsequiae.* Copenhagen: Hafniae: Petri Hakius; 1653.
20. Natale G, et al. Scholars and scientists in the history of the lymphatic system. *J Anat.* 2017;231(3):417-29.
21. Pizzi M, et al. A lucky mistake: the splenic glands of Marcello Malpighi. *Hum Pathol.* 2018;72:191-5.
22. Ijpma FFA, and van Gulik TM. "Anatomy Lesson of Frederik Ruysch" of 1670: A Tribute to Ruysch's Contributions to Lymphatic Anatomy. *World J Surg.* 2013;37(8):1996-2001.
23. Proulx ST, and Detmar M. Molecular mechanisms and imaging of lymphatic metastasis. *Exp Cell Res.* 2013;319(11):1611-7.
24. Di Matteo B, et al. Art in Science: Giovanni Paolo Mascagni and the Art of Anatomy. *Clin Orthop Relat Res.* 2015;473(3).
25. Schröer H. Relevance and reliability of Ludwig's scientific conceptions of the physiology of the microcirculation. *Pflugers Arch.* 1996;432(0031-6768 (Print)):R23-32.

26. Sappey PC. *Anatomie, physiologie, pathologie des vaisseaux lymphatiques considérés chez l'homme et les vertébrés. .* Paris: Delahaye; 1874.
27. Milroy WF. An Undescribed Variety of Hereditary Oedema. *NY Med J.* 1892;56:505-8.
28. Fine LG. Ernest Henry Starling (1866-1927) on the Formation and Reabsorption of Lymph. *Nephron Physiol.* 2014;126(3):9-17.
29. Meige H. Dystrophie Oedemateuse Hereditaire. *La Presse Medicale.* 1898;6:341-3.
30. Sabin FR. On the origin of the lymphatic system from the veins and the development of the lymph hearts and thoracic duct in the pig. *Am J Anat.* 1902;1(3):367-89.
31. Sabin F. *The Origin and Development of the Lymphatic System.* Baltimore: John Hopkins Press; 1913.
32. Braithwaite LR. The flow of lymph from the ileocæcal angle, and its possible bearing on the cause of duodenal and gastric ulcer. *Br J Surg.* 1923;11(41):7-26.
33. Drinker CK, et al. The Flow, Pressure, and Composition of Cardiac Lymph *Am J Physiol-Legacy Content.* 1940;130(1):43-55.
34. Warren MF, and Drinker CK. The Flow of Lymph from the Lungs of the Dog. *Am J Physiol-Legacy Content.* 1942;136(1):207-21.
35. Rouvière H. *Anatomy of the human lymphatic system.* Paris: Masson & Cle; 1938.
36. Patek PR. The morphology of the lymphactics of the mammalian heart. *Am J Anat.* 1939;64(2):203-49.
37. Kinmonth JB, Taylor, G.W. . Proceedings of the Physiological Society. *J Physiol.* 1956;133(suppl):3.
38. Heller A. On independent rhythmical contractions of lymph vessels in mammals. *Centralblatt für die medicinischen Wissenschaften* 1869;25(35):545-8.
39. Singhal D, et al. Technological Advances in Lymphatic Surgery: Bringing to Light the Invisible. *Plast Reconstr Surg.* 2019;143(1):283-93.
40. Kinmonth JB. Lymphangiography in man; a method of outlining lymphatic trunks at operation. *Clin Sci.* 1952;11(1):13-20.
41. Comparini L Fau - Bastianini A, and Bastianini A. Graphic reconstructions in the morphological study of the hepatic lymph vessels. *Angiologica.* 1965;2(0003-3189 (Print)):81-95.
42. Gould Ea Fau - Winship T, et al. Observations on a "sentinel node" in cancer of the parotid. *Cancer.* 1960 Jan-Feb;13(0008-543X (Print)):77-8.
43. Miller JFAP. IMMUNOLOGICAL FUNCTION OF THE THYMUS. *The Lancet.* 1961;278(7205):748-9.
44. Gowans JI Fau - Knight EJ, and Knight EJ. The Route of Re-circulation of Lymphocytes in the Rat *Proc R Soc Lond B Biol Sci.* 1964;14(0950-1193 (Print)):257-82.
45. Aprelikova O, et al. FLT4, a novel class III receptor tyrosine kinase in chromosome 5q33-qter. *Cancer Res* 1992 Feb 1;52(0008-5472 (Print)):756-48.
46. Joukov V, et al. Proteolytic processing regulates receptor specificity and activity of VEGF-C. *EMBO J.* 1997;16(13):3898-911.
47. Kukk E, et al. VEGF-C receptor binding and pattern of expression with VEGFR-3 suggests a role in lymphatic vascular development. *Development.* 1996;122(12):3829-37.
48. Joukov V, et al. Vascular endothelial growth factors VEGF-B and VEGF-C. *J Cell Physiol.* 1997;173(2):211-5.
49. Karpanen T, et al. Vascular Endothelial Growth Factor C Promotes Tumor Lymphangiogenesis and Intralymphatic Tumor Growth1. *Cancer Res.* 2001;61(5):1786-90.
50. Mandriota SJ, et al. Vascular endothelial growth factor-C-mediated lymphangiogenesis promotes tumour metastasis. *EMBO J.* 2001;20(4):672-82.

51. Mäkinen T, et al. Inhibition of lymphangiogenesis with resulting lymphedema in transgenic mice expressing soluble VEGF receptor-3. *Nat Med.* 2001;7(2):199-205.
52. Dumont DJ, et al. Cardiovascular Failure in Mouse Embryos Deficient in VEGF Receptor-3. *Science.* 1998;282(5390):946-9.
53. Mebius RE, et al. Developing Lymph Nodes Collect CD4+CD3− LTβ+ Cells That Can Differentiate to APC, NK Cells, and Follicular Cells but Not T or B Cells. *Immunity.* 1997;7(4):493-504.
54. Banerji S, et al. LYVE-1, a New Homologue of the CD44 Glycoprotein, Is a Lymph-specific Receptor for Hyaluronan. *J Cell Biol.* 1999;144(4):789-801.
55. Wigle JT, and Oliver G. Prox1 Function Is Required for the Development of the Murine Lymphatic System. *Cell.* 1999;98(6):769-78.
56. Breiteneder-Geleff S, et al. Angiosarcomas Express Mixed Endothelial Phenotypes of Blood and Lymphatic Capillaries: Podoplanin as a Specific Marker for Lymphatic Endothelium. *Am J Pathol.* 1999;154(2):385-94.
57. Skobe M, et al. Concurrent Induction of Lymphangiogenesis, Angiogenesis, and Macrophage Recruitment by Vascular Endothelial Growth Factor-C in Melanoma. *Am J Pathol.* 2001;159(3):893-903.
58. Skobe M, et al. Induction of tumor lymphangiogenesis by VEGF-C promotes breast cancer metastasis. *Nat Med.* 2001;7(2):192-8.
59. Suga K, et al. Visualization of Breast Lymphatic Pathways With an Indirect Computed Tomography Lymphography Using a Nonionic Monometric Contrast Medium Iopamidol: Preliminary Results. *Invest Radiol.* 2003;38(2).
60. Karkkainen MJ, et al. Vascular endothelial growth factor C is required for sprouting of the first lymphatic vessels from embryonic veins. *Nat Immunol.* 2004;5(1):74-80.
61. Srinivasan RS, et al. Lineage tracing demonstrates the venous origin of the mammalian lymphatic vasculature. *Genes Dev.* 2007;21(19):2422-32.
62. Ogata F, et al. Novel Lymphography Using Indocyanine Green Dye for Near-Infrared Fluorescence Labeling. *Ann Plast Surg.* 2007;58(6).
63. Ogata F, et al. Intraoperative Lymphography Using Indocyanine Green Dye for Near-Infrared Fluorescence Labeling in Lymphedema. *Ann Plast Surg.* 2007;59(2).
64. François M, et al. Sox18 induces development of the lymphatic vasculature in mice. *Nature.* 2008;456(7222):643-7.
65. . NIH Research Festival 2009. Symposium Lymphatic Biology and Disease: the Cinderella of the Vascular System Finally Gets Invited to the Ball. https://researchfestival.nih.gov/festival09/. Accessed 4/2/2023.
66. Nadolski GJ, and Itkin M. Feasibility of Ultrasound-guided Intranodal Lymphangiogram for Thoracic Duct Embolization. *J Vasc Interv Radiol.* 2012;23(5):613-6.
67. Yang L, et al. Evaluating glymphatic pathway function utilizing clinically relevant intrathecal infusion of CSF tracer. *J Transl Med.* 2013;11(1):107.
68. Ringstad G, et al. Glymphatic MRI in idiopathic normal pressure hydrocephalus. *Brain.* 2017;140(10):2691-705.
69. Aspelund A, et al. A dural lymphatic vascular system that drains brain interstitial fluid and macromolecules. *J Exp Med.* 2015;212(7):991-9.
70. Louveau A, et al. CNS lymphatic drainage and neuroinflammation are regulated by meningeal lymphatic vasculature. *Nat Neurosci.* 2018;21(10):1380-91.
71. Absinta M, et al. Human and nonhuman primate meninges harbor lymphatic vessels that can be visualized noninvasively by MRI. *eLife.* 2017;6:e29738.
72. Mahadevan A, et al. The Left-Right Pitx2 Pathway Drives Organ-Specific Arterial and Lymphatic Development in the Intestine. *Dev Cell.* 2014;31(6):690-706.
73. Martinez-Corral I, et al. Nonvenous Origin of Dermal Lymphatic Vasculature. *Circ Res.* 2015;116(10):1649-54.

74. Klotz L, et al. Cardiac lymphatics are heterogeneous in origin and respond to injury. *Nature.* 2015;522(7554):62-7.
75. Dori Y, et al. Dynamic Contrast-enhanced MR Lymphangiography: Feasibility Study in Swine. *Radiology.* 2014;273(2):410-6.
76. Itkin MG, et al. Diagnosis and Treatment of Lymphatic Plastic Bronchitis in Adults Using Advanced Lymphatic Imaging and Percutaneous Embolization. *Ann Am Thorac Soc.* 2016;13(10):1689-96.
77. Krishnamurthy R, et al. Imaging the Central Conducting Lymphatics: Initial Experience with Dynamic MR Lymphangiography. *Radiology.* 2014;274(3):871-8.
78. Dori Y, et al. Successful Treatment of Plastic Bronchitis by Selective Lymphatic Embolization in a Fontan Patient. *Pediatrics.* 2014;134(2):e590-e5.
79. Aspelund A, et al. The Schlemm's canal is a VEGF-C/VEGFR-3–responsive lymphatic-like vessel. *J Clin Invest.* 2014;124(9):3975-86.
80. Karpinich NO, and Caron KM. Schlemm's canal: more than meets the eye, lymphatics in disguise. *J Clin Invest.* 2014;124(9):3701-3.
81. Park D-Y, et al. Lymphatic regulator PROX1 determines Schlemm's canal integrity and identity. *J Clin Invest.* 2014;124(9):3960-74.
82. Truong TN, et al. Novel Characterization and Live Imaging of Schlemm's Canal Expressing Prox-1. *PLoS One.* 2014;9(5):e98245.
83. NIH. The Third Circulation: Lymphatics as Regulators in Health and Disease Workshop. https://videocast.nih.gov/watch=16750. Accessed 3/31/2023.
84. Regev A, et al. The Human Cell Atlas. *eLife.* 2017;6:e27041.
85. NIH. HuBMAP -The Human BioMolecular Atlas Program. https://hubmapconsortium.org/. Accessed March 28, 2023.
86. Snyder MP, et al. The human body at cellular resolution: the NIH Human Biomolecular Atlas Program. *Nature.* 2019;574(7777):187-92.
87. Lee H, et al. The Feasibility of Mesenteric Intranodal Lymphangiography: Its Clinical Application for Refractory Postoperative Chylous Ascites. *J Vasc Interv Radiol.* 2018;29(9):1290-2.
88. NHLBI. Yet to Be Charted: Mapping the Lymphatic System Across Body Scales and Expertise Domains Workshop. https://videocast.nih.gov/watch=43995. Accessed 3/31/2023.
89. Homayun-Sepehr N, et al. KRAS-driven model of Gorham-Stout disease effectively treated with trametinib. *JCI Insight.* 2021;6(15).
90. NHLBI. Yet to be Charted: Lymphatic System in Health and Disease. https://www.niddk.nih.gov/news/meetings-workshops/2022/yet-to-be-charted-lymphatic-system-in-health-and-disease. Accessed March 28, 2023.
91. NIH. The Research, Condition, and Disease Categorization Process. https://report.nih.gov/funding/categorical-spending/rcdc. Accessed March 28, 2023.
92. Congress tUS. https://docs.house.gov/billsthisweek/20220307/BILLS-117RCP35-JES-DIVISION-H_Part1.pdf; 2022:53.
93. Gale NW, et al. Angiopoietin-2 Is Required for Postnatal Angiogenesis and Lymphatic Patterning, and Only the Latter Role Is Rescued by Angiopoietin-1. *Dev Cell.* 2002;3(3):411-23.
94. Ortsäter H, et al. An inducible Cldn11-CreERT2 mouse line for selective targeting of lymphatic valves. *Genesis.* 2021;59(7-8):e23439.
95. Kriederman BM, et al. FOXC2 haploinsufficient mice are a model for human autosomal dominant lymphedema-distichiasis syndrome. *Hum Mol Genet.* 2003;12(10):1179-85.
96. Huang SS, et al. CRSBP-1/LYVE-l-null mice exhibit identifiable morphological and functional alterations of lymphatic capillary vessels. *FEBS Lett.* 2006;580(26):6259-68.

97. Gale NW, et al. Normal Lymphatic Development and Function in Mice Deficient for the Lymphatic Hyaluronan Receptor LYVE-1. *Mol Cell Biol.* 2007;27(2):595-604.
98. Connor AL, et al. Lymphatic endothelial lineage assemblage during corneal lymphangiogenesis. *Lab Invest.* 2016;96(3):270-82.
99. Gil HJ, et al. A novel podoplanin-GFPCre mouse strain for gene deletion in lymphatic endothelial cells. *Genesis.* 2018;56(4):e23102.
100. Wigle JT, et al. Prox1 function is crucial for mouse lens-fibre elongation. *Nat Genet.* 1999;21(3):318-22.
101. Hägerling R, et al. Intravital two-photon microscopy of lymphatic vessel development and function using a transgenic Prox1 promoter-directed mOrange2 reporter mouse. *Biochem Soc Trans.* 2011;39(6):1674-81.
102. Bazigou E, et al. Genes regulating lymphangiogenesis control venous valve formation and maintenance in mice. *J Clin Invest.* 2011;121(8):2984-92.
103. Choi I, et al. Visualization of lymphatic vessels by Prox1-promoter directed GFP reporter in a bacterial artificial chromosome-based transgenic mouse. *Blood.* 2011;117(1):362-5.
104. Truman LA, et al. ProxTom Lymphatic Vessel Reporter Mice Reveal Prox1 Expression in the Adrenal Medulla, Megakaryocytes, and Platelets. *Am J Pathol.* 2012;180(4):1715-25.
105. Bianchi R, et al. A Transgenic Prox1-Cre-tdTomato Reporter Mouse for Lymphatic Vessel Research. *PLoS One.* 2015;10(4):e0122976.
106. Zhu J, et al. Simultaneous in vivo imaging of blood and lymphatic vessel growth in Prox1–GFP/Flk1::myr–mCherry mice. *The FEBS Journal.* 2015;282(8):1458-67.
107. Kang GJ, et al. Intravital Imaging Reveals Dynamics of Lymphangiogenesis and Valvulogenesis. *Sci Rep.* 2016;6(1):19459.
108. Hong M, et al. Efficient Assessment of Developmental, Surgical and Pathological Lymphangiogenesis Using a Lymphatic Reporter Mouse and Its Embryonic Stem Cells. *PLoS One.* 2016;11(6):e0157126.
109. Jung E, et al. Development and Characterization of A Novel Prox1-EGFP Lymphatic and Schlemm's Canal Reporter Rat. *Sci Rep.* 2017;7(1):5577.
110. Scallan JP, et al. Foxo1 deletion promotes the growth of new lymphatic valves. *J Clin Invest.* 2021;131(14).
111. Baldwin ME, et al. Vascular Endothelial Growth Factor D Is Dispensable for Development of the Lymphatic System. *Mol Cell Biol.* 2005;25(6):2441-9.
112. Dellinger MT, et al. Vascular Endothelial Growth Factor Receptor-2 Promotes the Development of the Lymphatic Vasculature. *PLoS One.* 2013;8(9):e74686.
113. Iljin K, et al. VEGFR3 gene structure, regulatory region, and sequence polymorphisms. *FASEB J.* 2001 Apr;15(0892-6638 (Print)):1028-36.
114. Karkkainen MJ, et al. A model for gene therapy of human hereditary lymphedema. *Proc Natl Acad Sci U S A.* 2001;98(22):12677-82.
115. Calvo C-F, et al. Vascular endothelial growth factor receptor 3 directly regulates murine neurogenesis. *Genes Dev.* 2011;25(8):831-44.
116. Martínez-Corral I, et al. In vivo imaging of lymphatic vessels in development, wound healing, inflammation, and tumor metastasis. *Proc Natl Acad Sci U S A.* 2012;109(16):6223-8.
117. Redder E, et al. Vegfr3-tdTomato, a reporter mouse for microscopic visualization of lymphatic vessel by multiple modalities. *PLoS One.* 2021;16(9):e0249256.
118. Doh SJ, et al. Fluorescent reporter transgenic mice for in vivo live imaging of angiogenesis and lymphangiogenesis. *Angiogenesis.* 2018;21(4):677-98.
119. Shin WS, and Rockson SG. Animal Models for the Molecular and Mechanistic Study of Lymphatic Biology and Disease. *Ann N Y Acad Sci.* 2008;1131(1):50-74.
120. Harris NR, et al. The ebb and flow of cardiac lymphatics: a tidal wave of new discoveries. *Physiol Rev.* 2022;103(1):391-432.

121. Blake JA, et al. Mouse Genome Database (MGD): Knowledgebase for mouse–human comparative biology. *Nucleic Acids Res.* 2021;49(D1):D981-D7.
